# Freestanding flexible, pure and composite form of reduced graphene oxide paper for ammonia vapor sensing

**DOI:** 10.1038/s41598-019-45408-4

**Published:** 2019-06-19

**Authors:** D. Selvakumar, H. Sivaram, A. Alsalme, A. Alghamdi, R. Jayavel

**Affiliations:** 1 Centre for Nanoscience and Technology, Anna University, Chennai, 600025 India; 2 Department of Chemistry, College of Science, King Saud University, P.O. Box 2455, Riyadh, 11451 Saudi Arabia

**Keywords:** Environmental impact, Synthesis of graphene

## Abstract

Metal oxides based graphene nanocomposites were used for ammonia vapour sensing. The self-assembly process was adopted to prepare freestanding flexible pure rGO, CeO_2_-rGO and SnO_2_-rGO composite papers. The structural studies confirmed the formation of rGO composite papers. The ammonia vapor sensing was demonstrated using an impedance analyzer at different humidity levels as well as concentration. The CeO_2_-rGO composite paper achieved a sensitivity of 51.70 ± 1.2%, which was higher than that of pure rGO and SnO_2_-rGO composite paper. Both the surfaces (top and bottom) of the papers are active in efficiently sensing ammonia, which makes the present work unique. The results reveal that metal oxide/rGO papers can be effectively utilized in real time sensor application.

## Introduction

The major cause of air pollution is due to the pollutants emerging from dying, plastic, pesticides and fertilizer industries. Ammonia is a major source for all these industrial process, which is toxic, odor and very harmful to human health^1,2^. The gaseous or liquid form of ammonia can be detected commonly by using polymer analyzers, metal oxide sensors, catalytic detectors and optical detectors^3^. The ammonia sensing mechanism using polymer film follows irreversible and reversible processes and causes a change in conductivity affecting the sensitivity. Metal oxide based semiconductors were developed for ammonia sensing^4–8^, which has the limitation of low sensitivity. In order to overcome these difficulties, catalytic sensors were used to detect the ammonia. The change in gas concentration modifies the catalytic carrier concentration, thereby facilitating the sensing behavior^9^. Since the sensitivity was still low in the aforementioned method, the development of nanomaterials with high surface to volume ratio is indispensable, which results in high sensitivity, selectivity and stability^10,11^. The polymer-nanotubes/nanorods composites synthesized by template-based self-assembly process were used for effective sensing applications^12^. The clad modified fiber-optic gas sensors with Ce, Li and Al-doped nanocrystalline ZnO were developed for room temperature sensing of ammonia, methanol and ethanol^13^.

The conductivity of the sensor materials plays a major role and the materials used so far were less conductive resulting in poor performance. The highly conductive graphene oxide with large surface area possesses low noise and was used for the gas/vapor through adsorption^14^. Graphene based materials have also been used for gas/vapor sensing in diverse applications^15–17^. The SnO_2_-reduced graphene oxide based porous film has been reported to be an effective gas sensor material with tunable sensitivity through UV irradiation^18^. Various methods used for ammonia sensing include colorimetric sensing^19^, conductometric sensing^20^, electrochemical sensing^21^, direct detection of ammonium ion by means of oxygen electrocatalysis^22^, resistance based sensing^23–25^, impedance analysis^26^ and Keithley electrometer sensing^27^. The gas sensing by impedance analysis is simple and compact experimental set-up compared with other methods.

The present work mainly focuses on the development of pure and composite form of freestanding flexible rGO paper as an efficient sensing material. The freestanding flexible, pure rGO, SnO_2_-rGO and CeO_2_-rGO composite based papers were prepared by self-assembly process. The ammonia vapor sensing was systematically studied for all samples. The flexible paper based materials do not require any holder or substrate, because of the freestanding nature. The ammonia molecules interact with both the active surfaces (top and bottom) of the rGO paper, enhancing the sensitivity, repeatability and process controllability.

## Materials and Method

The pure and composite form of rGO papers were prepared by using self-assembly method as reported earlier^28–31^. In order to improve the sensor performance, tin oxide (SnO_2_) and cerium oxide (CeO_2_) were separately incorporated into the rGO paper to make the composite structure. The cerium(III) chloride heptahydrate (10 wt%) was mixed with the as-synthesized graphene oxide powder and motorized for 30 min followed by sonication for 3 h to form the CeO_2_-rGO composite gel. By the same way, SnO_2_-rGO composite gel was also prepared. The gels were poured over the silane coated petri-plate and dried at 60 °C. The pure and composite form of GO papers were prepared and annealed at 400 °C under argon atmosphere to get reduced GO (rGO) paper. The as-prepared freestanding flexible rGO papers were sintered to enhance the conductivity and then directly subjected to gas sensing application.

### Characterization

The crystallinity of pure and composite form of rGO papers was studied by powder XRD analysis with Cu-Kα radiation using RIGAKU MINIFLEX II-C system. Raman spectra were recorded using HORIBA JOBIN YVON LABRAM HR micro Raman system with excitation wavelength of 488 nm of the argon-ion laser. The morphology of prepared samples was studied using TESCAN VEGA 3 SBU Scanning Electron Microscopy (SEM) and the elemental mapping by FEI Quanta FEG 200 High-Resolution SEM. TEM images were obtained with LA D6 source in TECNAI T-30 HRTEM. The gas sensing properties were analyzed by using WEYNE KERR 6500B precision impedance analyzer. Functional groups were identified before and after exposure of ammonia using FT/IR-6300 type A FTIR instrument with ATR PRO470-H mode.

## Results and Discussion

**Figure 1:**
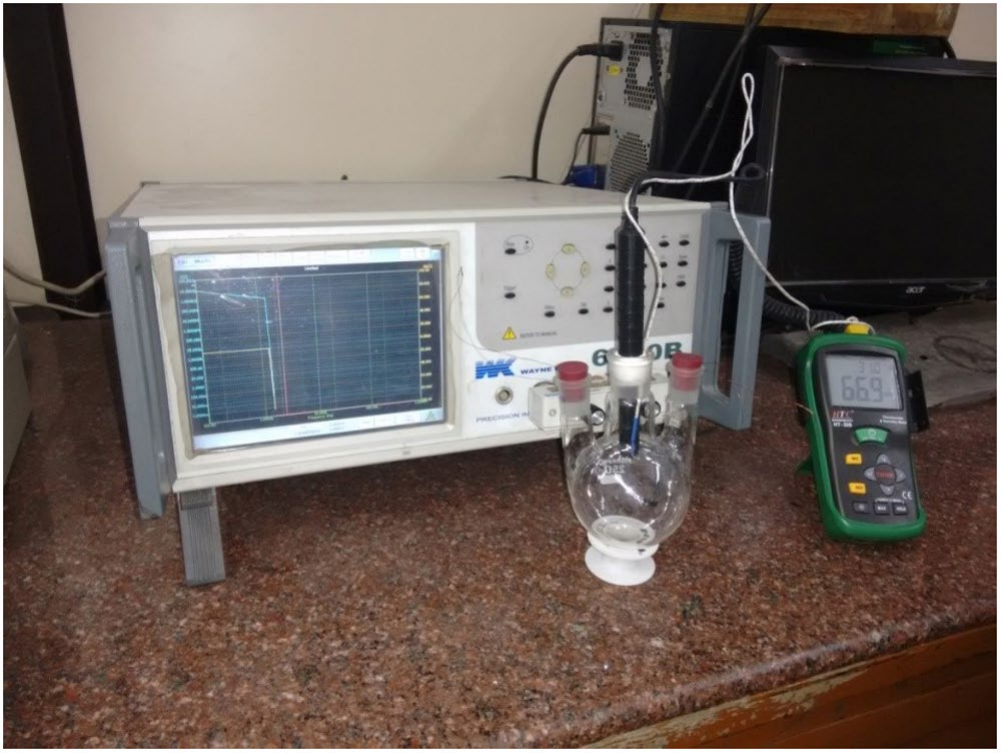
Experimental set-up designed for ammonia sensing using metal oxide-rGO composites.

Figure 1 shows the experimental set-up for ammonia sensing with the sample size of 1 cm^2^ loaded into the flask. The electrical contacts were made on both the edges of the sample directly connected to the impedance analyzer. The ac voltage was applied as an input to the sample and the change-in electrical impedance was measured with respect to ammonia vapor as a function of relative humidity and concentration. The electrical signal varies on the exposure of ammonia vapor at various humidity and concentration levels. The ammonia vapor sensing in the present study was realized with a simple and cost effective experimental set-up.

**Figure 2:**
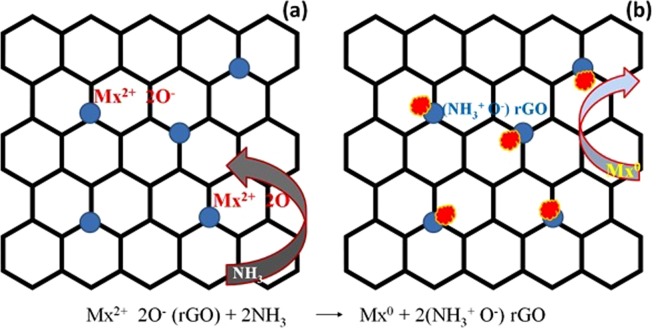
Proposed mechanism of ammonia detection by the metal oxide-rGO composites.

The mechanism of ammonia detection is given by Eq. (1)

1 $${{\rm{Mx}}}^{2+}2{{\rm{O}}}^{-}({\rm{rGO}})+2{{\rm{NH}}}_{3}\to {\rm{Mx}}^\circ +2({{{\rm{NH}}}_{3}}^{+}{{\rm{O}}}^{-}){\rm{rGO}}$$

where, Mx is the metal oxide. The metal oxide is used as a catalyst to enhance the sensing of gases^32^. The rGO sheet possesses mesoporous structure used as a carrier for metal oxides, which adsorbs large amount of toxic gases. Ammonia possesses electron donating nature and it can be directly absorbed by metal oxide complexes. In addition, the reversible process takes place in ammonia by proton transfer phenomenon. Figure 2 shows the adsorption of ammonia on the surface of the composite structures. It is to be noted that the ammonia can directly link to the composite materials without altering the crystalline structure. Figure 2(a) represents the situation before the exposure of ammonia to the rGO composite paper. After exposure of ammonia, it can be directly adsorbed on to the composite materials as shown in Fig. 2(b). The structure of pure and composite form of rGO paper remains unaltered after the exposure of ammonia.

**Figure 3:**
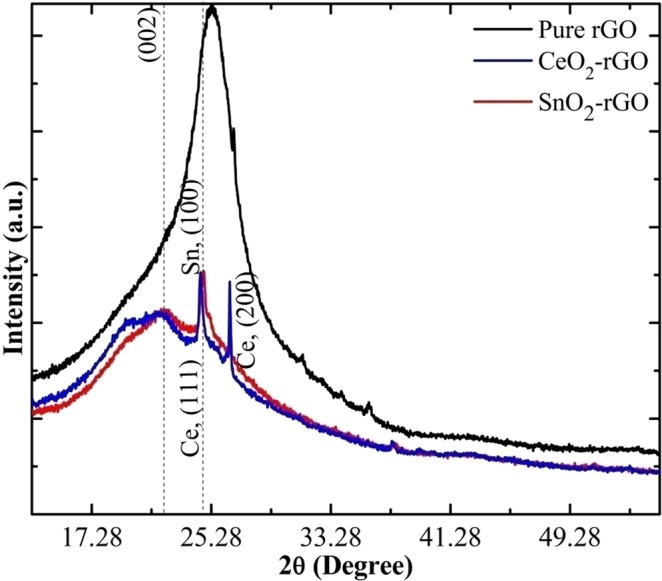
XRD patterns of pure rGO, CeO_2_-rGO and SnO_2_-rGO composite papers.

Powder XRD patterns of pure and composite form of rGO papers are shown in Fig. 3. The intensity of the XRD pattern decreases for composite sample confirming the incorporation of SnO_2_/CeO_2_ materials onto the rGO paper. The broad peak at 2θ = 25.06° confirms the formation of pure rGO paper corresponding to the (002) orientation. For the metal oxide/rGO composite, the (002) rGO peak is very broad in the 2θ range 21–25°. The peak at 2θ = 24.82° represents the (100) plane of SnO_2_ and the peaks at 24.53° and 26.53° correspond to CeO_2_ in the rGO matrix. The sharp peaks of Sn and Ce appear to be overlapping with the broad rGO peak and hence the peak corresponding to rGO is not very prominent in the composite. The interlayer spacing was calculated to be 3.51, 4.028 and 3.982 Å for pure rGO, CeO_2_-rGO and SnO_2_-rGO composites respectively.

**Figure 4:**
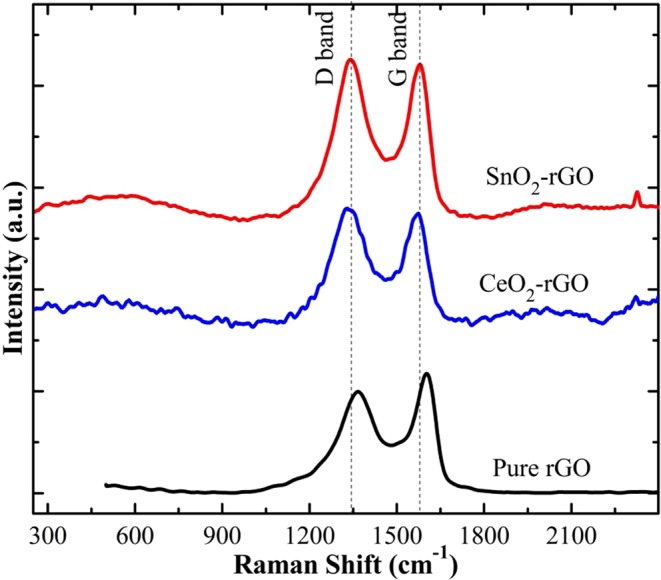
Raman spectra of pure and composite form of rGO papers.

Raman spectra of pure and composite form of rGO papers are shown in Fig. 4. The G band at 1359 cm^−1^ and D band at 1601 cm^−1^ confirm the formation of pure rGO paper. The G and D bands of SnO_2_-rGO composite paper was observed at 1573.52 cm^−1^ and 1336.06 cm^−1^. The CeO_2_-rGO composite paper possesses G band at 1571.93 cm^−1^ and D band at 1335.17 cm^−1^. Due to the addition of SnO_2_/CeO_2_ in rGO matrix_,_ the band position is shifted to lower wavenumber. The I_D_/I_G_ ratio of pure rGO paper was calculated to be 0.85 and increased to 1.008 for SnO_2_-rGO composite paper and 1.022 for CeO_2_-rGO composite paper. The incorporation of metal oxides creates more defects in rGO paper, thereby increasing the D band intensity for composite samples^33^.

**Figure 5:**
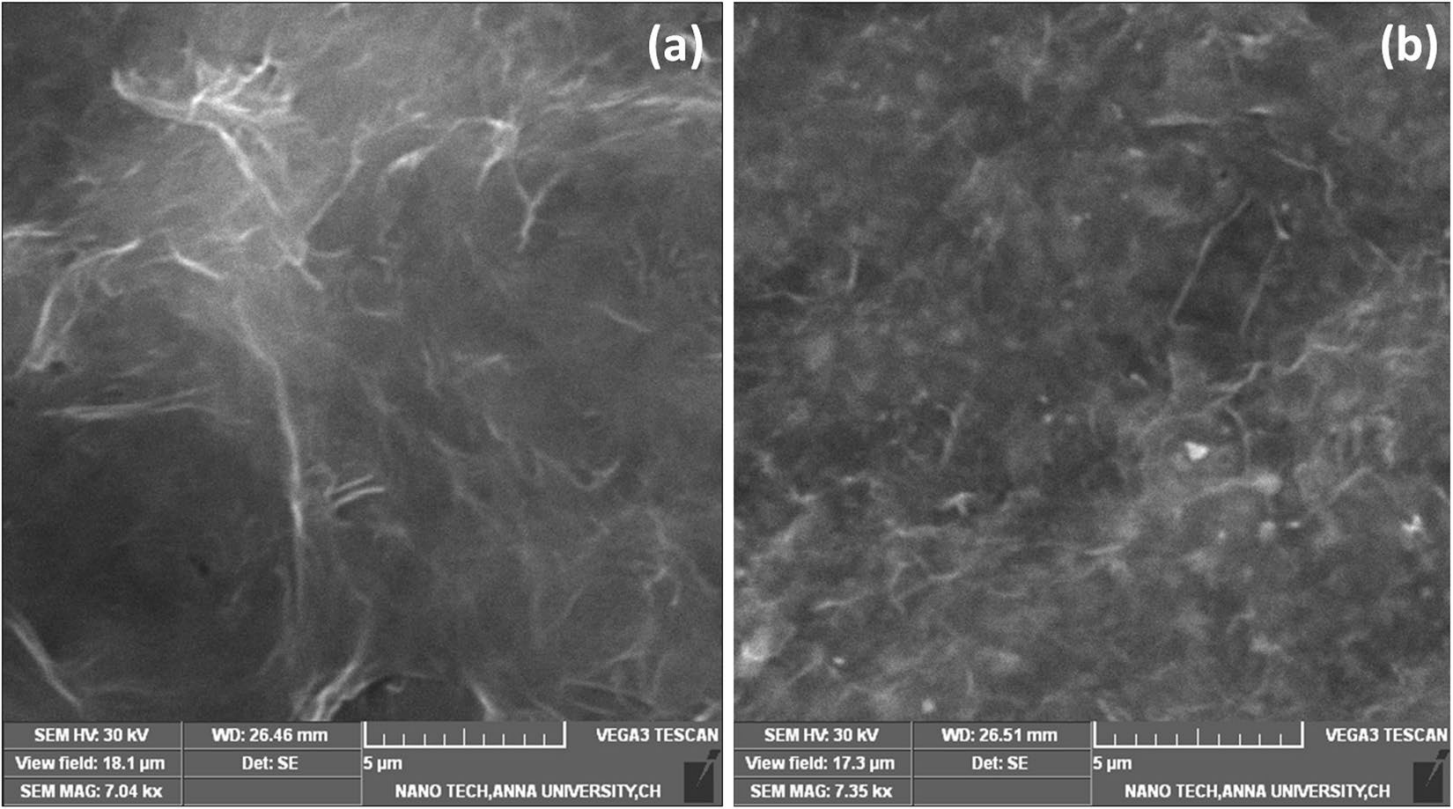
SEM images of (**a**) SnO_2_-rGO and (**b**) CeO_2_-rGO composite papers.

**Figure 6:**
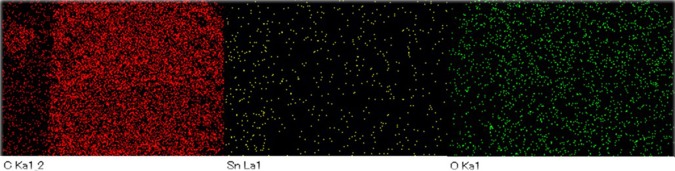
Elemental mapping of SnO_2_-rGO composite paper.

**Figure 7:**
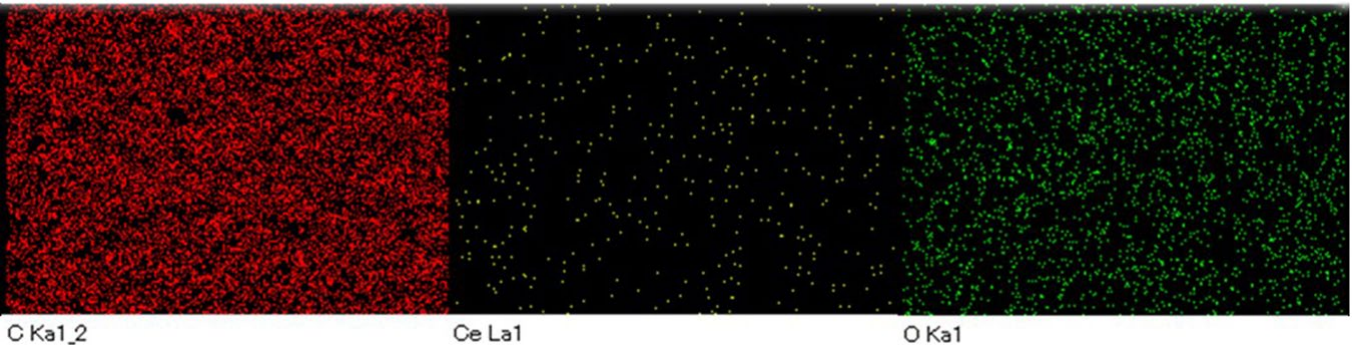
Elemental mapping of CeO_2_-rGO composite paper.

**Figure 8:**
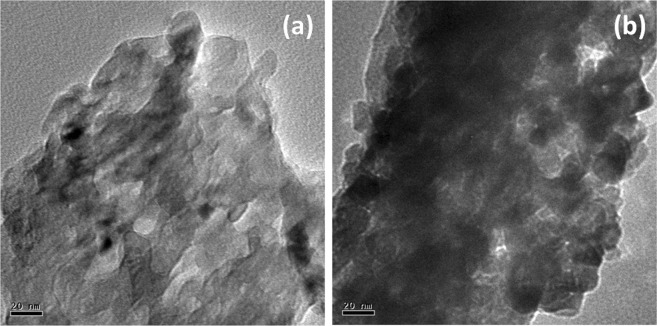
HRTEM image of (**a**) SnO_2_-rGO and (**b**) CeO_2_-rGO composite papers.

The morphology of SnO_2_-rGO and CeO_2_-rGO composite papers are shown in Fig. 5. The sheet-like morphology was observed in both the cases. The addition of 10 wt% of SnO_2_ does not influence the morphology SnO_2_-rGO composite paper. The CeO_2_ particles adhered to the rGO composite were clearly identified through the SEM image shown in Fig. 5(b). The elemental mapping of SnO_2_-rGO and CeO_2_-rGO composite papers shown in Figs 6 and 7 confirms the composition as well as the distribution of individual elements. HRTEM image of SnO_2_-rGO and CeO_2_-rGO composite papers are shown in Fig. 8. The metal oxide particles were distributed over the rGO paper as confirmed in the SEM images. Figure 8(a) shows agglomerated SnO_2_ particles distributed over the rGO paper, whereas in CeO_2_-rGO composite paper, well-disbursed CeO_2_ particles are uniformly distributed over the rGO paper (Fig. 8(b)), which enhances the ability of ammonia sensing.

**Figure 9:**
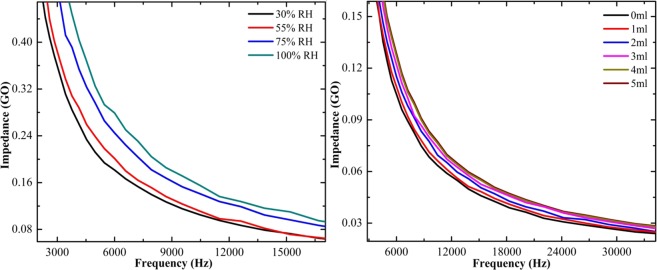
Ammonia vapor sensing of pure rGO paper at different levels of relative humidity and concentration.

**Figure 10:**
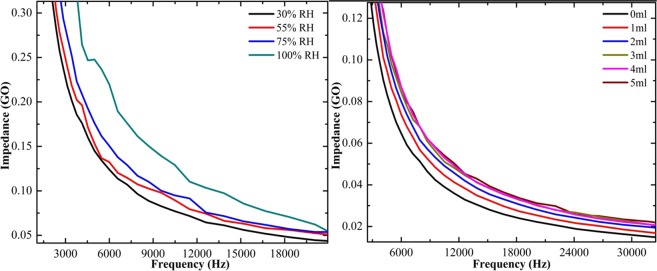
Ammonia vapor sensing of SnO_2_-rGO composite paper at different levels of relative humidity and concentration.

**Figure 11:**
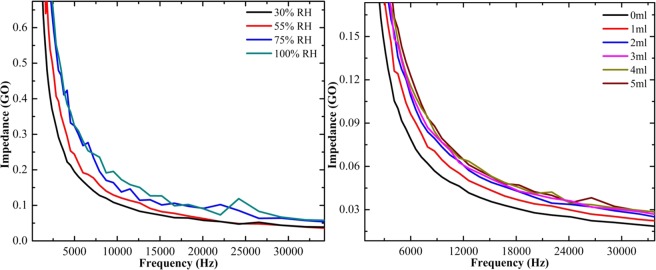
Ammonia vapor sensing of CeO_2_-rGO composite paper at different levels of relative humidity and concentration.

The exposure of ammonia vapor over the pure and composite form of rGO papers was detected directly by the impedance analyzer. Figure 9 shows the ammonia vapor sensing of pure rGO paper under different relative humidity (RH) and concentration levels. The change in impedance with respect to the applied frequency is plotted. The increase in impedance value with increasing relative humidity confirms the adsorption of ammonia over rGO paper at different humidity levels. The affinity of ammonia is the maximum at higher humidity level, resulting in higher impedance value. The ammonia sensing was also measured by varying the concentration of ammonia. The plot shows that while increasing the concentration, the impedance gradually increases and saturated at 3 mL for pure rGO paper. The ammonia sensing for SnO_2_-rGO composite paper shows that the impedance value increased by increasing the relative humidity and concentration as shown in Fig. 10. Initially, the impedance increased slowly with increasing humidity level and there was a drastic change in the impedance for 100% humidity. While increasing the concentration, the impedance value increased for SnO_2_-rGO composite paper and saturated at 3 mL of ammonia. Similarly, the sensing of CeO_2_-rGO composite paper was analyzed as shown in Fig. 11. It also shows impedance increases with increasing humidity and concentration. The sensitivity of the ammonia adsorbed over the sample was calculated using Eq. (2)

2 $${\rm{S}}=({{\rm{Z}}}_{{\rm{final}}}-{{\rm{Z}}}_{{\rm{initial}}}){/Z}_{{\rm{initial}}}$$

where, Z_final_ and Z_initial_ are the final and initial concentration of the ammonia taken.

The sensitivity of pure rGO paper was calculated to be 23.28 ± 0.7%. The sensitivity of the SnO_2_-rGO composite paper was 41.04 ± 1.0% and for the CeO_2_-rGO composite paper the sensitivity was 51.70 ± 1.2%. The semiconducting SnO_2_ has n-type conductivity with a band gap of around 3.3–4.3 eV based on the type of synthesis process, which limits the possibility of higher chemisorption of NH_3_ molecules. Ce doping meritoriously influences the electronic properties of rGO matrix, which can be explained by the following equation: The general sensing reaction can be represented as

$${{\rm{O}}}_{2({\rm{gas}})}+{\rm{e}}\to {{{\rm{O}}}_{2}}^{-}$$

$$4{{\rm{NH}}}_{3}+5{{{\rm{O}}}_{2}}^{-}\to 4{\rm{NO}}+6{{\rm{H}}}_{2}{\rm{O}}+5{{\rm{e}}}^{-}$$

$${{\rm{CeO}}}_{2}\to {{\rm{Ce}}}_{{\rm{Y}}}\,\cdot \cdot \,+2{{{\rm{O}}}^{{\rm{x}}}}_{{\rm{o}}}+2{\rm{e}}-;\,{\rm{Y}}={\rm{rGO}}\,{\rm{matrix}}$$

The Ce^4+^ ions doped into rGO matrix, which binds with graphene to form the electron-donor defects (CeY∙∙). The electron neutrality is maintained by the release of two electrons into the conduction band by Ce ions to increase the response^34^. When the sensor is exposed to ammonia gas, chemisorbed NH_3_ molecules react with the superoxide (O_2_^−^) molecule of CeO_2_ and change the sensor resistance. Moreover, the cubic structure of CeO_2_ can easily engrave with graphene matrix, enhancing the catalytic activity of CeO_2_-rGO material thereby sensing the toxic gases more effectively compared to SnO_2,_ which is less efficient for ammonia sensing.

The experiments were performed thrice over a period of 60 days. After each experiment, the material was kept at 400 °C in a tubular furnace to remove the ammonia completely. The sensitivity showed a variation of about ±1.2% in each cycle. The long-term stability was tested over 60 days.

Table 1 summarizes the different methods used for ammonia sensing with various materials. The polymer materials showed poor sensitivity and bulk nanomaterials were developed to maximize the sensitivity. Carbon based materials (carbon nanotubes and graphene) were developed to enhance the sensing response. In the present work, the metal oxide were embedded/connected to the reduced graphene oxide matrix resulting in a sensing response of 51.70 ± 1.2% for CeO_2_-rGO composite paper, which is comparable to the other methods reported earlier. The homemade set-up is used for ammonia sensing, due to its simple operation protocol and low cost. The present sensors are of freestanding, flexible in nature and both the surface can be used for ammonia sensing applications.

**Table 1:** Figure of merits of ammonia sensing by different methods.

Material	Synthesis method	NH_3_ sensing method	Sensitivity	Reference
Colloidal silica beads modified with quantum dots and zinc (II) tetraphenylporphyrin	Assembling monodisperse silica nanoparticles via a microfluidic device	Colorimetric sensing	7 ppm	^19^
Mesoporous TiO_2_ beads functionalized with gold nanoparticles (AuNPs-TiO_2_)	Structure-directing template assisted growth	Conductometric sensing	5.56 over 600 ppm (Ethanol)	^20^
Silver oxide nanoparticle decorated carbon nanotubes (Ag_2_O/CNT NCs)	Wet chemical method	Electrochemical sensing	32.856 μAμM^-1^ cm^−2^	^21^
TiO_2_/GO/PANI	*In-situ* oxidative chemical polymerization	Conductivity mode	5 ppm	^11^
Polyaniline ultrathin layers on reduced graphene oxide sheets	*In-situ* polymerization method	Chemiresistive Sensing	3.0 × 10^−4^/ppm for 100 ppm	^17^
TiO_2_:Ce nanoparticles	Sol–gel method	Impedance Analysis	2.5 for 500 ppm at 175 °C	^26^
TiO_2_/SnO_2_/WO_3_ hybrid nanostructures	Ultra-sonic assisted Sol–gel wet impregnation method	Keithley Electrometer	59.90%	^27^
PPy/NDSA and PPy/DBSA layers	*In-situ* chemical deposition	Quartz Crystal Microbalance	4 ppm	^9^
CeO_2_-rGO composite paper	Self-assembly method	Impedance Analysis	51.70 ± 1.2%	Present work

## Conclusion

This is the first demonstration of the freestanding flexible, pure rGO, SnO_2_-rGO and CeO_2_-rGO composite papers prepared by a self-assembly process used for ammonia sensing. The structural and morphology of pure and composite rGO papers were confirmed by XRD, SEM and HRTEM analysis. The Raman spectrum and EDX analysis confirmed the incorporation of SnO_2_ and CeO_2_ in rGO matrix. The change in electrical signal upon the exposure of ammonia at different relative humidity as well as concentration confirmed the sensing property of ammonia with simple cost effective impedance analyzer at room temperature. The CeO_2_-rGO composite paper exhibited higher sensitivity (51.70 ± 1.2%) against various concentration of ammonia. The ammonia sensing mechanism has also been explored and the result highlights that the CeO_2_-rGO composite paper is a promising candidate for real time ammonia leakage monitoring applications.

## Supplementary information

Freestanding flexible, pure and composite form of reduced graphene oxide paper for ammonia vapor sensing
