# Supplementary material for: Freestanding flexible, pure and composite form of reduced graphene oxide paper for ammonia vapor sensing
Source: Sci Rep. 2019 Jun 19;9:8749. doi: 10.1038/s41598-019-45408-4 (PMC6584526; doi:10.1038/s41598-019-45408-4)
Supplement: Supplementary file 1 — Freestanding flexible, pure and composite form of reduced graphene oxide paper for ammonia vapor sensing [file 41598_2019_45408_MOESM1_ESM.pdf]

Supplementary Information

Freestanding flexible, pure and composite form of reduced graphene oxide paper for ammonia vapor sensing

D. Selvakumar[a], H. Sivaram[a], A. Alsalme[b], A. Alghamdi[b], R. Jayavel[a,*]

[a] Centre for Nanoscience and Technology, Anna University, Chennai – 600025, India.

[b] Department of Chemistry, College of Science, King Saud University, P.O. Box 2455, Riyadh 11451, Saudi Arabia.

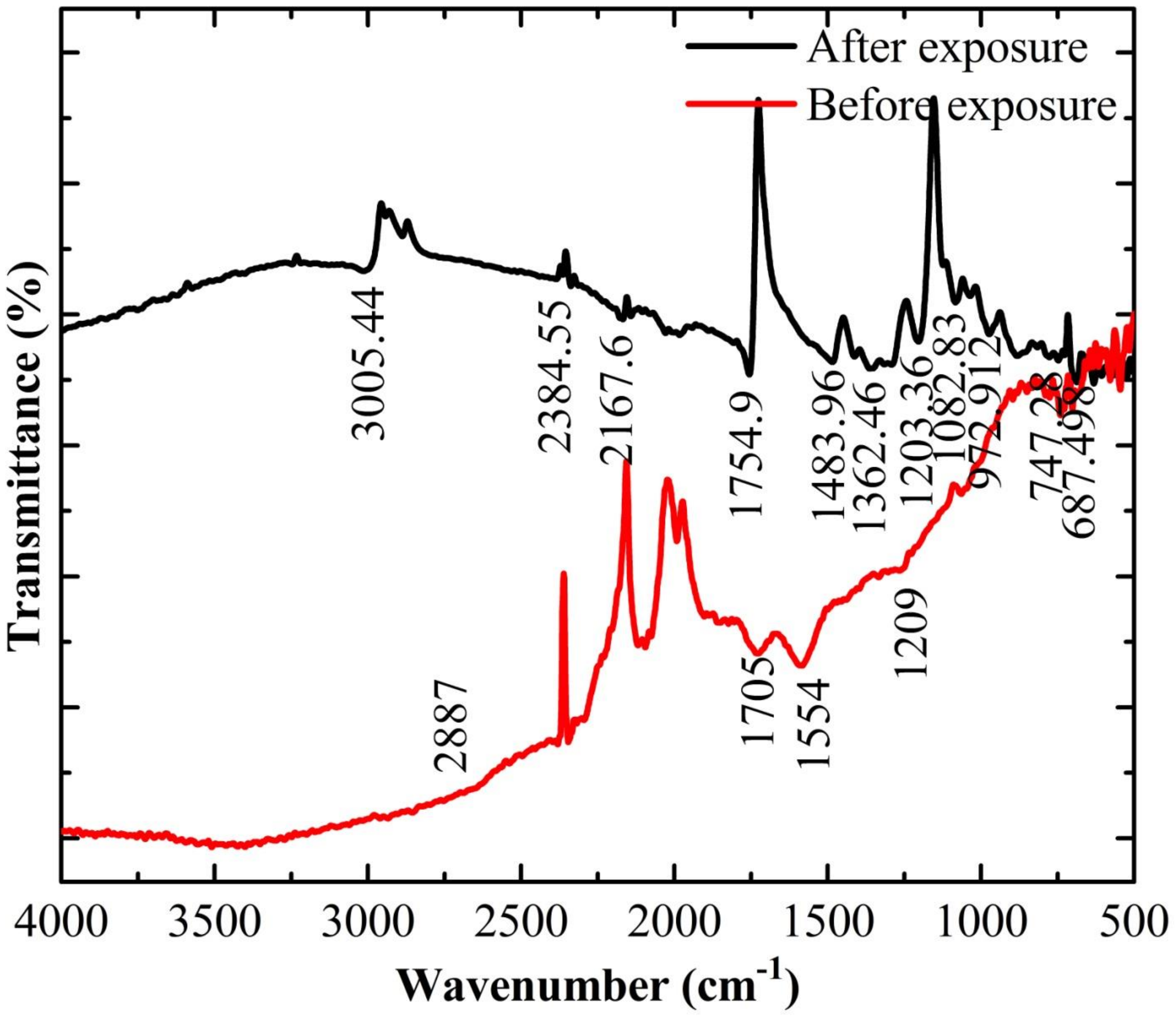

Figure S1: FTIR spectra of pure rGO paper before and after ammonia exposure

The FTIR spectra of pure rGO paper before and after exposure of ammonia was shown in Figure S1. The vibration spots of before exposure are marked as that of our previous reports. After exposure of ammonia to the pure sample the adsorption of ammino functional groups [1] –N=C=N– stretching vibration at 2167.6 cm^{-1}, N–H bending vibration at 1483.96 cm^{-1}, C–N stretching vibration at 1082.83 cm^{-1} and N–N stretching vibration at 972.12 cm^{-1}. The FTIR spectra of CeO_2-rGO paper before and after ammonia exposure of ammonia shown in Figure S2. The presence of ceria adsorps more amount of ammonia and the major peaks corresponds to ammonia shifts to higher wavenumber in the given figure. The FTIR spectra of SnO_2-rGO paper before and after ammonia exposure of ammonia shown in Figure S3. The tin oxide doped sample

gives the similar kind of adsorption as that of pure rGO paper. The peaks corresponds to the functional groups are marked in the FTIR spectra.

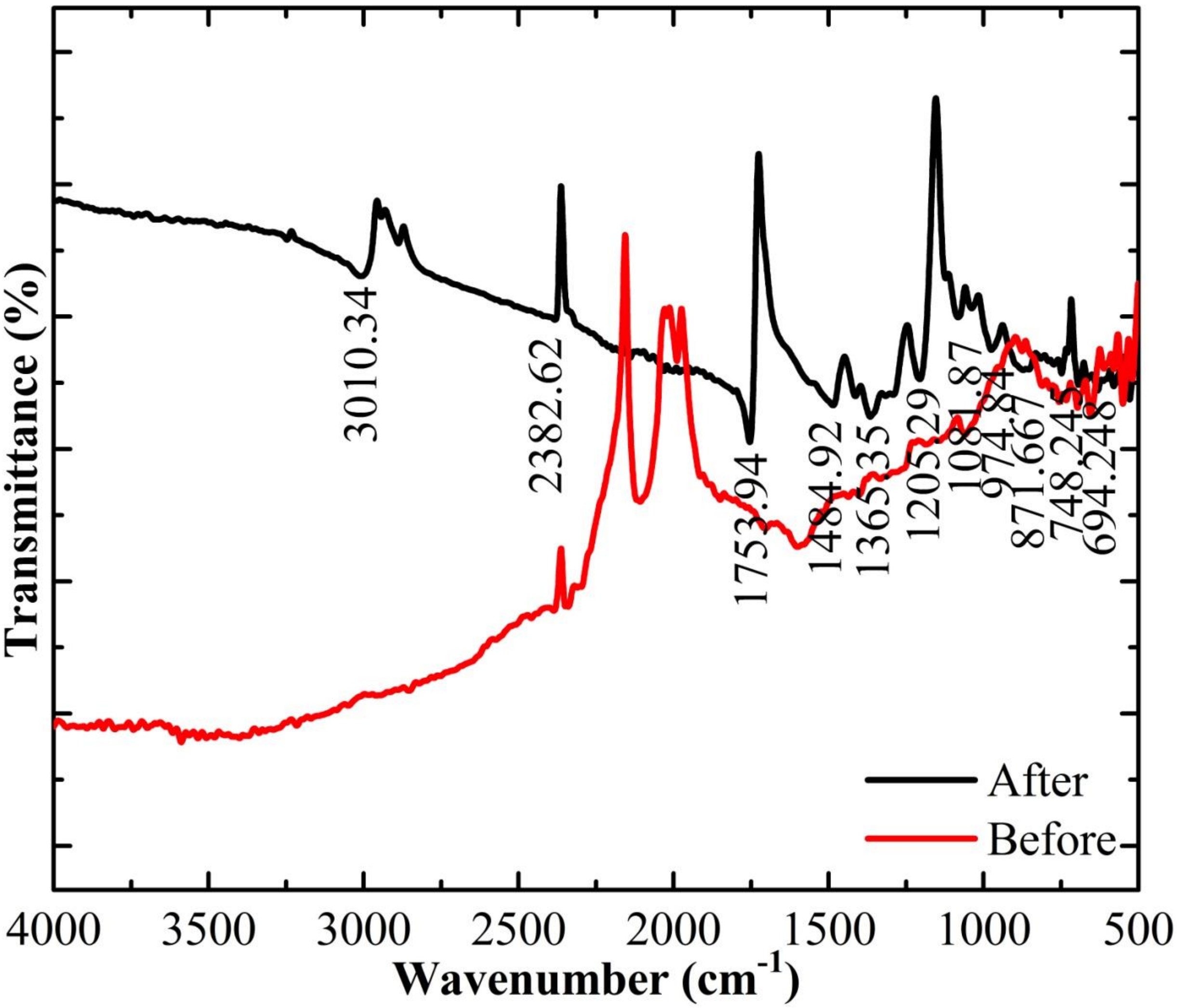

Figure S2: FTIR spectra of CeO_2-rGO paper before and after ammonia exposure

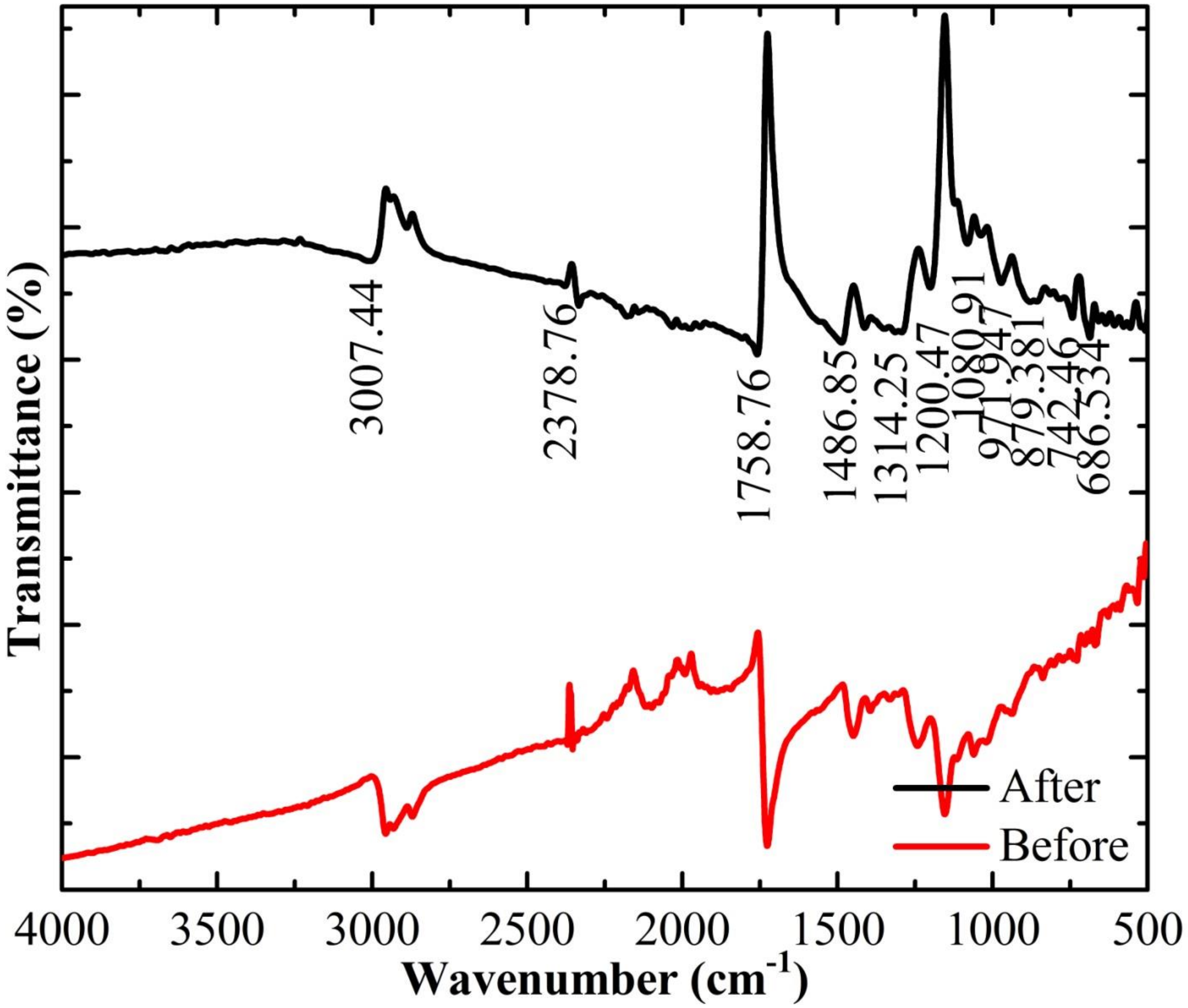

Figure S3: FTIR spectra of SnO_2-rGO paper before and after ammonia exposure

Reference

[1] T.F. Tahir, A. Salhin and S.A. Ghani, Flow Injection Analysis of Mercury Using 4-Dimethylamino) Benzaldehyde-4-Ethylthiosemicarbazone as the Ionophore of a Coated Wire Electrode, Sensors **2012**, 12, 14968-14982.
